# Mixed mania associated with cessation of breastfeeding

**DOI:** 10.1186/s40345-016-0059-z

**Published:** 2016-09-05

**Authors:** Kristen A. Schmidt, Brian A. Palmer, Mark A. Frye

**Affiliations:** Department of Psychiatry and Psychology, Mayo Clinic, 200 1st Street SW, Rochester, MN 55905 USA

**Keywords:** Bipolar disorder, Breastfeeding, Weaning, Mania

## Abstract

**Background:**

This case chronicles the unique presentation of psychotic mixed mania in a female 5 months after parturition and 1 week following breastfeeding discontinuation, highlighting a rarely recognized mania risk factor that is temporally delayed from parturition: breastfeeding discontinuation.

**Case presentation:**

A 25-year-old G1P1 female with a past psychiatric history of a depressive episode in adolescence presented to the Emergency Department with her 5-month-old daughter, fiancée, and family 1 week after breastfeeding cessation. She endorsed sleep-deprived energy enhancement, unfulfilled goal-oriented productivity, hyper-talkativeness, hyper-sexuality and increased nicotine use. Concurrent depressive symptoms included hopelessness, worthlessness, poor concentration, lack of appetite, and ego-dystonic intrusive thoughts that she may kill herself or her child. She exhibited pressured speech, affective lability, expansiveness, distractibility, and tangential, grandiose, delusional self-referential content. Transient thoughts of self-harm and harm to her child were not associated with intent. Her family history was significant for a deceased mother who had bipolar I disorder. The patient was hospitalized for 5 days and diagnosed with bipolar disorder, type I, current episode manic with psychotic features with a mixed-feature specifier. Olanzapine and lithium were initiated and the patient’s acute episode of mania resolved prior to discharge.

**Conclusions:**

This case extends the limited literature on mania following weaning and highlights the role of rapid serum dopamine rise following breastfeeding cessation in mania.

## Background

Breastfeeding involves a positive feedback loop whereby prolactin surges stimulate alveolar milk production and reduce tonic dopamine inhibition (Andrews et al. 2001; Lyons et al. 2012; Plotsky and Neill 1982; Romano et al. 2013; World Health Organization 2009). The soporific effects of prolactin and oxytocin facilitate rest after nighttime nursing (World Health Organization 2009). Breastfeeding cessation disrupts biorhythms and dopamine quiescence (Lyons et al. 2012; Romano et al. 2013; World Health Organization 2009) which may have profound consequences for mothers predisposed toward affective illness.

## Case presentation

A 25-year-old G1P1 female presented to the Emergency Department in March, 2015, with her 5-month-old daughter, fiancée, and concerned family members 1 week after breastfeeding cessation.

Despite 3 years of sustained sobriety from opioid use, she reported a de novo obsession about relapsing to heroin when her fiancée received an opiate prescription. After attending multiple AA meetings and three consecutive nights without sleep, she believed the big book of alcoholics anonymous was talking to her for a special purpose. She reported sleep-deprived energy enhancement, unfulfilled goal-oriented productivity, hyper-talkativeness, hyper-sexuality and increased nicotine use. Concurrent depressive symptoms included hopelessness, worthlessness, poor concentration, lack of appetite, and ego-dystonic intrusive thoughts that she may kill herself or her child. She reported daily cannabis and cigarette (10/day) use since parturition and was not on any medications and did not consume alcohol. Cannabis use was chronic and she had never experienced a psychotic episode prior to breastfeeding discontinuation. Her psychiatric history included a major depressive episode at age 16 treated briefly with citalopram and an opioid use disorder in full sustained remission for 3 years. Her family history included a mother with bipolar I disorder.

Her mental status was significant for pressured speech, affective lability, expansiveness, distractibility, and tangential, grandiose, delusional self-referential content. Her transient thoughts of self-harm and harming her child were not associated with intent. Physical examination revealed lower abdominal tenderness commensurate with her first menses since parturition. Urine drug screen was positive for cannabis.

Symptoms remitted during a 5-day hospitalization with olanzapine and lithium. Her discharge diagnosis was bipolar disorder, type I, current episode manic with psychotic features and a mixed-feature specifier.

## Discussion

This is a case of psychotic mixed mania 5-month after parturition in a patient with one prior major depressive episode and a family history of bipolar I disorder. While there is particular awareness of psychoses in the immediate post-partum period related to changes in estrogen/progesterone levels in combination with sleep deprivation, this case highlights a psychosis risk factor that is temporally delayed from parturition and related to a second set of neuroendocrine factors: breastfeeding discontinuation-associated acute mania.

Related case literature is limited. Joyce et al. (1981) reported on a patient who developed mania after weaning both of her children. Xu et al. (2014) found the first psychiatric hospitalization in bipolar patients occurred 1 month later for breastfeeding than for non-breastfeeding mothers post-parturition. While sleep disruption due to infant care and the psychosocial stresses associated with new parenting may contribute to manic induction, oxytocin and prolactin have intrinsic sleep promoting properties and their waning during weaning may affect maternal sleep wake cycles (World Health Organization 2009).

The primary neuroendocrine basis for breastfeeding discontinuation-associated acute mania likely relates to dopaminergic changes during breastfeeding. In a non-lactating state, dopamine inhibits prolactin release through anterior pituitary lactotrophs expressing D2 receptors (Andrews et al. 2001; Lyons et al. 2012; Plotsky and Neill 1982; Romano et al. 2013). During lactation, however, positive feedback from suckling increases prolactin, and dopamine’s tone is markedly reduced (Andrews et al. 2001; Plotsky and Neill 1982; Romano et al. 2013). This may relate to prolactin’s impact on tyrosine hydroxylase, the rate-limiting agent in dopamine synthesis, which influences dopamine downregulation during breastfeeding (Andrews et al. 2001; Romano et al. 2013).

Nursing naturally reduces dopamine, which is a pharmacotherapeutic intervention exploited by most effective mania treatments (Cookson et al. 1982). Early studies examining the neuroendocrine system and mania observed rising serum prolactin levels following neuroleptic administration with manic symptom improvement, supporting the conclusion that dopaminergic overactivity is involved in mania pathogenesis (Cookson et al. 1982).

## Conclusion

The case we report extends the limited literature on mania following weaning (Xu et al. 2014), and it addresses an oft-forgotten source of rapid serum dopamine rise: the cessation of breastfeeding.
